# “We are the sun for our community:” Partnering with community health workers/promotores to adapt, deliver and evaluate a home-based collaborative care model to improve equity in access to quality depression care for older U.S. Latino adults who are underserved

**DOI:** 10.3389/fpubh.2023.1079319

**Published:** 2023-02-02

**Authors:** Lesley E. Steinman, Amelia Gasca, Theresa J. Hoeft, Patrick J. Raue, Stuart Henderson, Rosa Perez, Alfredo Huerta, Alex Fajardo, Melinda A. Vredevoogd, Katherine James, Ladson Hinton, Laura Rath, Jurgen Unutzer

**Affiliations:** ^1^Department of Health Systems and Population Health, Health Promotion Research Center, School of Public Health, University of Washington, Seattle, WA, United States; ^2^El Sol Neighborhood Educational Center, San Bernardino, CA, United States; ^3^Department of Psychiatry and Behavioral Sciences, School of Medicine, University of Washington, Seattle, WA, United States; ^4^School of Medicine Office of Research, University of California Davis, Sacramento, Sacramento, CA, United States; ^5^Department of Psychiatry and Behavioral Sciences, University of California Davis, Sacramento, Sacramento, CA, United States; ^6^Archstone Foundation, Long Beach, CA, United States

**Keywords:** collaborative care, depression, Latino, health equity, implementation science, adaptations, community health workers, older adults

## Abstract

**Background:**

While depression is a leading cause of poor health, less than half of older adults receive adequate care. Inequities in both access and outcomes are even more pronounced for socially disadvantaged older adults. The collaborative care model (CCM) has potential to reduce this burden through community-based organizations (CBOs) who serve these populations. However, CCM has been understudied in diverse cultural and resource-constrained contexts. We evaluated the implementation and effectiveness of PEARLS, a home-based CCM adapted with and for community health workers/promotores (CHWs/Ps).

**Methods:**

We used an instrumental case study design. Our case definition is a community-academic partnership to build CHW/P capacity for evidence-based depression care for older U.S. Latino adults in the Inland Empire region of California (2017–2020). We aimed to understand adaptations to fit local context; acceptability, feasibility, and fidelity; clinical effectiveness; and contextual determinants of implementation success or failure. Data sources included quantitative and qualitative administrative and evaluation data from participants and providers. We used descriptive statistics and paired *t*-tests to characterize care delivery and evaluate effectiveness post-intervention, and deductive thematic analysis to answer other aims.

**Findings:**

This case study included 152 PEARLS participants and nine data sources (*N* = 67 documents). The CBO including their CHWs/Ps partnered with the external implementation team made adaptations to PEARLS content, context, and implementation strategies to support CHWs/Ps and older adults. PEARLS was acceptable, feasible and delivered with fidelity. Participants showed significant reductions in depression severity at 5 months (98% clinical response rate [mean (SD), 13.7 (3.9) drop in pre/post PHQ-9; *p* < 0.001] and received support for 2.6 social needs on average. PEARLS delivery was facilitated by its relative advantage, adaptability, and trialability; the team's collective efficacy, buy-in, alignment with organization mission, and ongoing reflection and evaluation during implementation. Delivery was challenged by weak partnerships with clinics for participant referral, engagement, reimbursement, and sustainability post-grant funding.

**Discussion:**

This case study used existing data to learn how home-based CCM was adapted by and for CHWs/Ps to reduce health inequities in late-life depression and depression care among older Latino immigrants. The CBOs and CHWs/Ps strong trust and rapport, addressing social and health needs alongside depression care, and regular internal and external coaching and consultation, appeared to drive successful implementation and effectiveness.

## 1. Introduction

Depression is a significant public health issue affecting one in four adults and is now the leading cause of health related disability (1). Among older persons, depression impairs function and quality of life, leads to worse health outcomes, and increases risk of preventable deaths including suicide (2). Older persons with depression use more health care (3–5) and enter nursing homes earlier (6). These institutionalizations can be devastating as most older persons prefer aging in place, and costs of health and social care continue to rise without improvement in health outcomes (7–9).

Despite depression's impact on people, society, and systems, and the existence of effective depression care models, there is a depression treatment gap around the world. Half of older adults do not receive adequate or appropriate treatment (10). Socially marginalized older persons have greater disparities in depression outcomes. Older persons living in poverty have higher burden of depression, worse access to care, and are less likely to benefit from pharmacological or psychotherapy treatment than older adults overall (11). Older persons of color have less access to sufficient care despite recent rises in both recognition and treatment rates (12–14), and those with limited English proficiency (LEP) are similarly disadvantaged (15). Persons with intersecting identities further increase their mental health burden (15). Even when care is available for underserved communities, it is typically poor quality and not evidence-based (16).

The global mental health field has emerged to address inequities in resource-constrained settings both domestically in the U.S. and internationally (17). There is an urgent call for closing the mental health treatment gap by building capacity among non-specialist workers tasks to deliver community-based interventions (1, 18). This task-shifting and—sharing can improve access to quality mental health care by expanding availability of self- and community-based care in settings with limited access, workforce gaps, and stigma toward specialty mental health care (19–21). Community-based care can also address negative social determinants of health like poor housing and food insecurity (22). While global mental health research was initially focused in resource-constrained settings outside the U.S. (“low- and middle-income countries”), a global-to-local frame is now called for to improve equity in access to appropriate, high-quality mental health care in resource-constrained contexts in high-income countries (HICs) (20, 23, 24).

The Program to Encourage Active, Rewarding Lives (PEARLS) is a home/community-based collaborative care model (CCM) created in partnership with social service organizations in Seattle, U.S. (25). The collaborative care model is a promising approach for improving access to high-quality and culturally appropriate late-life depression care. CCM builds capacity among non-mental health providers to provide effective team-based care (26). While initially developed for highly-resourced clinical settings (27, 28), encouraging evidence is emerging in resource-constrained settings (29–32). Research has shown that older person's financial, physical, and cultural barriers to care can be reduced by offering preferred non-pharmacological care (33) in their homes (34). PEARLS' focus on marginalized older persons can also improve health equity (35) through organizations that address social determinants of health (34, 36, 37).

Training CHWs/Ps to provide PEARLS has potential to reduce mental health inequities faced by older underserved communities of color. Also known as lay health workers, lay providers, *promotoras de salud*, and other terms, CHWs/Ps are a “frontline public health worker who is a trusted member of and/or has a close understanding of the community served. This trusting relationship enables the worker to serve as a liaison/link/intermediary between health/social services and the community to facilitate access to services and improve the quality and cultural competence of service delivery (38).” CHWs/Ps also build capacity within the community through increasing health knowledge and self-sufficiency *via* outreach, education, informal counseling, offering social support, and advocacy. A recent systematic review of the literature (39) found CHW/P-delivered mental health care was effective at improving mental health outcomes for underserved communities. This review, however, found evidence lacking for CHWs/Ps delivering evidence-based programs (EBPs) as the primary care provider in resource-constrained settings in the U.S. Furthermore, there were limited details about implementation strategies needed to support CHWs/Ps to provide mental health EBPs.

This know-do gap is a focus for implementation science: a research and practice approach to understand what, why, and how interventions work in “real world” settings and evaluate ways to improve them (40, 41). One reason this gap persists is that EBPs typically come from academic settings, leading to models that need to be adapted to more resource constrained contexts (42–44). Implementation science for health equity recognizes the need to adapt EBPs for local context to improve both delivery and outcomes, and doing this adaptation in partnership with, for and in communities (44–46). The purpose of this case study is to evaluate the implementation and effectiveness of adapted CHW/P-delivered PEARLS for older depressed Latino immigrants. Our specific research questions (RQ) are:

1. What adaptations were made to PEARLS intervention and implementation strategies to fit the local context, and how and why were these adaptations made?
2. What were the contextual determinants of PEARLS implementation with older Latino immigrants?
3. Was PEARLS acceptable, feasible, done with fidelity, and sustainable (implementation outcomes)?
4. How clinically effective was PEARLS for older Latino immigrants?

## 2. Methods

### 2.1. Design

We used an instrumental case study design (47) to understand how adapting PEARLS for delivery with CHWs/Ps impacted depression care delivery and outcomes. We used Hyett et al. (48) case study methodology to guide both study execution and manuscript preparation. This study was determined to be exempt by the University of Washington Institutional Review Board.

### 2.2. Case definition and selection

Our case definition is a community-academic partnership to build capacity for collaborative care between 2017 and 2020 (49). We used Kohrt's et al. (22) recommendations for mapping community-based mental health care to generate the case description: Our partnership included two applied research centers at the University of Washington (UW) and a social service community-based organization (El Sol Neighborhood Educational Center; “El Sol”) that trains *promotores de salud* [community health workers/promotores (CHWs/Ps)] to improve access to and quality of social service care for underserved communities in the Inland Empire region of California. El Sol, a CBO committed to community transformation and social change, prioritizes reaching mono-lingual Spanish speakers, immigrants, and other LEP residents. Two CHWs/Ps were trained to deliver PEARLS to older adults in their homes. The depression care management (implementation) team included the CHWs/Ps, a program manager/CHW/P supervisor at the community-based organization, and a licensed mental health therapist and a psychiatric assistant at local partner organizations. The research center role was to provide content and quality improvement expertise *via* practice coaching (“external facilitation”) (50) and project oversight. PEARLS participants were primarily Spanish-speaking Mexican-American immigrants who had lived in the U.S. for over 10 years. These older adults were living in poverty with multiple chronic conditions and poor access to quality health care. PEARLS participant engagement occurred through healthy aging presentations at low-income housing, social service agencies, and health care organizations, where participants received fresh fruit and vegetable boxes and completed brief depression screening to assess eligibility.

### 2.3. Intervention

**Figure 1:**
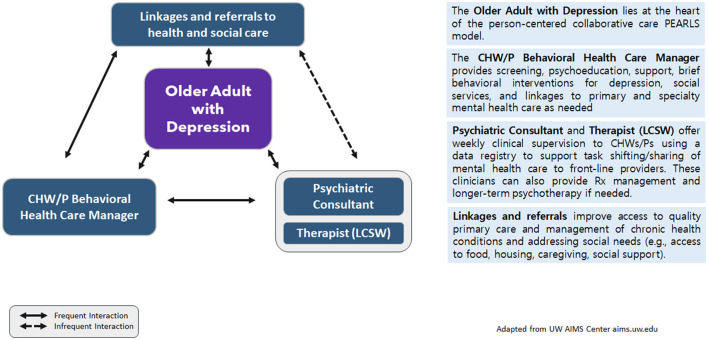
Model for adaptation design and impact: modified for PEARLS *via* CHWs/Ps for older Latino adults^a^. ^a^Modified from Kirk et al. (55) MADI framework.

PEARLS is a home-based collaborative care model. Older adults living with depression are at the heart of this person-centered care model. A front-line provider serves as the behavioral health care manager—they are trained to actively screen for and monitor depression using the Patient Health Questionnaire (PHQ-9) (51); offer brief psychosocial interventions [Problem-Solving Treatment (52), Behavioral Activation (53)] for self-management, support, and psychoeducation; and linkages to address social needs and coordinate with primary and mental health care as needed. External clinical supervisors provide ongoing consultation and training for non-mental health specialists to provide quality depression care. Based on the Chronic Care Model (54), PEARLS empowers older persons to manage depression and often other chronic conditions, using existing resources, new skills, and better care linkages. While in clinic-based collaborative care, the primary care provider (PCP) is part of providing team-based care, in community- or home-based collaborative care, the community-based organization engages older adults from multiple clinical care providers. As such, improving access to culturally and linguistically appropriate and affordable quality health care becomes part of the linkages provided during program delivery. A summary of the PEARLS intervention used for this case study is provided in Figure 1 and will be described in further detail in the Findings section on adaptations.

### 2.4. Data sources

We used routine PEARLS process and outcome data from several sources (Table 1). An electronic data registry included participant data from the 152 older adults who engaged in PEARLS from August 2017 to July 2020. This registry included quantitative data on our primary outcome of interest [depression severity, response and remission as measured by the PHQ-9 to evaluate clinical effectiveness (RQ4). The registry also included qualitative data on referrals for social and health needs. We categorized these social needs referrals using several social determinants of health frameworks (56–60) to create a comprehensive list. Registry data also included quantitative process data on screening, enrollment, completion, and average number of home and phone sessions for implementation fidelity (RQ3).

**Table 1:** Case study data sources.

**Data source**	**Sample size**
Qualitative data **(*****N*** = **67)**	
Interview text data	•Participant testimonials (*n* = 9) •Provider interviews (*n* = 8)
Implementation documents	•Notes from technical assistance/coaching (*n* = 16) calls and sustainability planning calls (*n* = 8) with the PEARLS implementation team and external practice coaches •Notes from clinical supervision calls (*n* = 4) •Intervention materials (*n* = 6) •Dissemination materials (*n* = 5) •Planning documents (*n* = 4) •Evaluation reports (*n* = 5)
Other materials	•Progress reports (*n* = 6)
Quantitative data **(*****N*** = **152)**	
Electronic data registry	PEARLS participants •PHQ-9 screening and assessment •PEARLS enrollment and completion •Number and mode of PEARLS sessions •Social needs referrals

We also reviewed nine qualitative data sources (67 documents) for the case study: (a) semi-structured interview transcripts with the PEARLS implementation team at two time points during program delivery; (b) participan*t test*imonials gathered post program completion; (c) *month*ly technical assistance/coaching calls notes with the PEARLS implementation team and external practice coaches; (d) sustainability planning coaching call notes with the program manager and director at the community-based organization, and external practice coaches; (e) clinical supervision session fidelity reports; (f) narrative progress reports; (g) intervention materials; (h) internal planning documents; (i) dissemination products; and (j) evaluation reports. These documents provided data to answer our four research questions on adaptations, determinants, implementation outcomes, and effectiveness.

### 2.5. Data analysis

We conducted a secondary data analysis using these routine program and evaluation data sources (2017–2020). We used descriptive statistics to report PEARLS participant characteristics and effectiveness (RQ4) under pragmatic conditions (61). We used paired *t*-tests to assess whether changes between baseline and post-PEARLS PHQ-9 (depressive symptom) scores were statistically significant. We also compared clinically significant changes in depression over time: response (% of participants with >/=50% reduction between pre- and post-PHQ-9) and remission (% with PHQ-9 <5). Lastly, we described PHQ-9 changes by demographic subgroups (e.g., age, gender). These were descriptive rather than statistical comparisons given small cell sizes in each group. We also summarized unintended benefits and consequences from participants and providers perspectives using the secondary qualitative data sources.

We addressed the other research questions *via* thematic analysis (62) of the 67 qualitative data sources (a) through (f)—using implementation science and cultural adaptations frameworks for deductive coding of adaptations, determinants, and implementation outcomes (Table 2), and inductive coding generating codes from the data to describe different partners' perspectives on program effectiveness. We used Gale and colleagues' rapid framework method (68) to organize the data by code, data source, and stakeholder, and generate initial themes. Thematic analysis consisted of six iterative phases to yield a rich, complex interpretation of the data: familiarization, generating codes, searching for, reviewing, defining and naming themes, and producing the report (69). LS led the analysis, with input from the implementation, coaching and evaluation team members on codebook development and refinement; describing, refining, and summarizing key themes; and interpretation of findings and implications. We used several recommended strategies from Lincoln and Guba (70) to strengthen the trustworthiness of our research (Table 3). To protect confidentiality, we do not identify the specific roles of the PEARLS implementation team in the findings.

**Table 2:** Theoretical implementation science and cultural adaptation frameworks used for deductive coding.

**Construct**	**Framework**	**Description**
Adaptations^a^	Model for adaptation design and impact (MADI) (55) expanded Framework for reporting adaptations and modifications to evidence-based interventions (FRAME) (44) Cultural influences on mental health framework (63) Heuristic framework for cultural adaptations of interventions (64)	•What adaptations were made •Who participated in adaptation decision-making •For whom the adaptation was made •When the adaptation occurred during the implementation process •Why, how, and under what circumstances adaptations were made •Culture influences meanings and norms about both depression and depression care through symptom expression; assessment and diagnosis; coping styles and help seeking; and treatment preferences •Partner with delivery organizations to specify when and how to do cultural adaptations
Determinants	Consolidated framework for implementation research (CFIR) (65) adapted for LMIC (66)	Facilitators and barriers to PEARLS delivery in six areas: •Intervention characteristics •Inner setting •Outer setting •Characteristics of individuals •Implementation process •System of care
Implementation outcomes	Implementation outcomes framework (67)	From the perspective of PEARLS participant, providers, and other partners •Acceptability (satisfaction) •Feasibility (actual fit) •Fidelity (faithfulness to core functions) •Sustainability (maintenance of program delivery)

a Adaptations to PEARLS, both intervention and implementation strategies, including cultural adaptations to improve both engagement in care and depression outcomes.

**Table 3:** Strengthening the trustworthiness of this research (70).

**Criteria**	**Definition (71)**	**Operationalization**
Credibility	How congruent are findings with reality? How well do the findings hang together?	•Prolonged engagement—engaging with the site and the data over the life cycle of PEARLS implementation •Data triangulation—using different data sources that include diverse stakeholder perspectives to identify patterns •Peer debriefing—gathering reactions on methods and findings from coresearchers and colleagues who are less involved in PEARLS delivery or evaluation •Member checking—providing initial findings to participants with multiple roles to provide feedback on accuracy
Transferability	How well do these findings apply to other contexts?	•Thick descriptions—of contextual information about the setting, providers, and participants in PEARLS delivery and evaluation
Dependability	How well do we trust the research methods, findings and interpretation	•Documentation of the research process including reflexive auditing to describe decisions made throughout the study •Peer debriefing—gathering reactions on methods and findings from coresearchers and colleagues who are less involved in PEARLS delivery or evaluation
Confirmability	How close to objective reality is our research?	•Use of audit trails and a reflexivity journal

## 3. Findings

PEARLS participants are described in Table 4. Participants ranged in age from 65 to over 80 years old (mean age 73). Two-thirds (69%) identified as female and the majority identified as Spanish-speaking (88%) Latino (91%). Other case study participants included care managers (CHWs/Ps), clinical supervisors, leadership (i.e., program manager/CHW/P supervisor and El Sol director), and practice coaches. Data sources are summarized in Table 1.

**Table 4:** PEARLS participant demographics (*N* = 152).

	***n* (%)**
**Age**	
65–69	50 (32.7)
70–74	32 (20.8)
75–79	31 (20.1)
80+	40 (26.4)
**Gender**	
Male	47 (30.9)
Female	105 (69.1)
**Race**	
American Indian/Alaska native	0 (0.0)
Asian	2 (1.3)
Black	4 (2.7)
Pacific Islander	0 (0.0)
White	144 (94.7)
Mixed race	2 (1.3)
**Ethnicity** ^a^	
Hispanic or Latino	138 (90.8)
Not hispanic or Latino	14 (9.2)
**Preferred language**	
English	18 (11.8)
Spanish	134 (88.2)

a We did not collect data on country of origin or immigration status. The CBO serves primarily monolingual Spanish-speaking Mexican-Americans who have immigrated to the U.S. within the past 10 years, though country of origin and time in the U.S. may vary.

**Figure 2:**
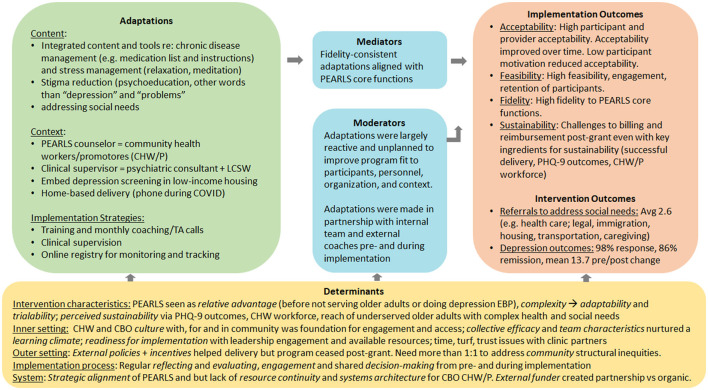
PEARLS intervention (home-based collaborative care) with community health workers/promotores/promotores (CHWs/Ps).

Figure 2 brings together key findings from each of the research questions to illustrate how PEARLS was adapted for impact.

### 3.1. Adaptations (RQ1)

Our first research question asks what adaptations were made to PEARLS intervention and implementation strategies to fit the local context, and how and why these adaptations were made. Most of the secondary data described what was adapted, including intervention content, delivery context, evaluation and training, implementation strategies, cultural adaptations, and adaptations for equity. Unless otherwise noted, these adaptations were fidelity-consistent and reactive to issues that needed to be addressed to improve implementation and effectiveness. Adaptations occurred during the implementation process among the El Sol team (CHWs/Ps trained as PEARLS care managers, their program manager/supervisor, and the clinical supervisors which included a licensed mental health therapist (LCSW) and a psychiatric consultant from a local partner organization) and at times in conversation with external practice coaches or other implementation supports. These adaptations did not alter the core functions of the intervention or implementation strategies but rather supported different forms for carrying out these functions.

#### 3.1.1. Context

Adaptation to context includes the personnel, population, format, and setting in which the intervention is delivered. For personnel, the community-based organization identified two CHWs/Ps to serve as PEARLS care managers. Training trusted CHWs/Ps who were well-known, live in, and know the community and culture was essential to engage older Latino immigrants in a depression care intervention, given the poor access to quality clinical care and stigma about both depression and depression care among this population. As a member of the PEARLS implementation team stated, “*We understand that a lot of these people, you know, are afraid to speak up. They don't know their rights. They don't know what they're able to do. So, we like being that voice for them and advocating for them, and empowering them because we're just passionate, you know? That's our passion is to work with people in our community* (IW_2019_A).

PEARLS complemented the CHWs/Ps scope of practice to provide culturally and linguistically appropriate education, counseling and support, and connect people to health and social care where people live, to address health disparities in both access to care and health outcomes (not all CHWs/Ps in the U.S. do home visits or are from the community where they work). Both a licensed mental health therapist and psychiatric consultant served as clinical supervisors, an adaptation from the original PEARLS model of a psychiatrist providing regular clinical supervision. The therapist was available to provide one-on-one therapy during or after PEARLS if needed, while the team's use of a psychiatric assistant offered a more affordable and accessible model than a psychiatrist.

#### 3.1.2. Content

The CHWs/Ps and community-based organization drove other adaptations to PEARLS. The primary modification to content was to address social needs both within and outside of PEARLS usual tools. Many of their older Latino population faced multiple intersecting social determinants of health that created inequities in both burden of disease (depression, loneliness, diabetes, hypertension) and access to care (both primary care and in-home caregiving). The primary social needs included poor-quality health care, lack of family or social support (in particular challenges with adult children), housing instability, limited access to legal, immigration and citizenship services, lack of quality in-home caregiving, dearth of transportation, and disconnect from spiritual or faith groups. Table 5 shares a breakdown of the 393 social needs referrals by type, of which 80% resulted in connection to services. The PEARLS team used both existing PEARLS tools (psychoeducation and monitoring of depression symptoms, problem-solving treatment, behavioral activation, and connection to clinical and social supports) as well as additional outreach and advocacy to connect underserved older adults to necessary services.

“*Even though we're focused on helping our clients, our patients develop skills to solve problems so that they can resolve their depression, many other issues come up along the way. Multiple medical issues might come up. Social issues, family conflict, isolation. So, pretty soon, we find out there's a lot more things going on than just depression. So, we find ourselves working with our clients to develop those skills to solve depression, but also we do a lot of advocacy….So, there's a lot of things that are going on aside from the strictly PEARLS-related work—we do a lot of advocacy and outreach for our clients. Which we see it as part of the—we cannot just focus on the PEARLS Program. We have to focus also on other issues ‘cause we know that's gonna help ‘em as well to resolve their depression. Because it's going to help improve their quality of life*” (IW_2019_D).

**Table 5:** PEARLS process of care: sessions, health education, social needs referrals (*N* = 149)^a^.

	***n* (%)**
**PEARLS sessions**	
Total contacts (phone or in-person)	1,467
Contacts, *mean (sd)*	*9.8 (2.0)*
Completed at least 6 PEARLS sessions	149 (100)
PEARLS sessions: home visits, *mean (sd)*	*6.6 (2.1)*
PEARLS sessions: phone visits, *mean (sd)*	*2.9 (0.9)*
% at least 1 psychiatric consult completed	109 (73.2)
**Health education materials**	***N*** = **246**
Stress, relaxation, self-care	69 (28.0)^b^
Chronic disease self-management	57 (23.2)
Sleep hygiene	40 (16.3)
Physical activity/exercise	38 (15.4)
Brain health/dementia	22 (8.9)
Mental health and substance abuse	10 (4.1)
Other (e.g., safe driving)	10 (4.1)
**Referrals to address social needs**	
Total # of referrals, *n*	393
Referrals connected to services	315 (80.1)
Referrals not applicable as already in services	42 (10.7)
Referrals not connected to services	36 (9.2)
**Referral type** ^c^	
Medical care including dental	75 (19.1)
Family/social support	53 (13.5)
Housing	36 (9.2)
Legal	31 (7.9)
Citizenship, immigration, ESL classes	27 (6.9)
In-home support services/caregiving	27 (6.9)
Transportation	26 (6.6)
Spiritual	25 (6.4)
Mental health	18 (4.6)
Food insecurity	17 (4.3)
Financial including utilities	16 (4.1)
Health insurance	14 (3.6)
Personal living/safety	12 (3.1)
Employment	10 (2.5)
Health information/education	6 (1.5)

a Participation data is for people with at least one session following enrollment (149/152, 98%).

b % of health information and education referrals made (N = 246).

c Referrals with less than 1%: dental care, employment, substance abuse.

All data are presented as *n* (%) except when displayed in italics which denotes data is presented as mean (sd).

In addition, the CHWs/Ps worked with their team to create additional tools to support client health needs and build skills while addressing gaps in care. These included Spanish-language medical packets for chronic disease management to track medications and primary care visits; activity packets with relaxation and breathing exercises and games for meaningful activities they could do from their home (bed or chair) and help manage daily stress; and sleep hygiene tips. Table 3 provides a breakdown of these health education materials (*N* = 246) that were shared with clients. CHWs/Ps modified the PEARLS activity lists to be tailored to a person's PHQ-9 symptoms, which facilitated the choice of activities that directly addressed symptoms that were bothering them. The program manager describes the need for and process for creating the medical packets.

“*The CHWs/Ps did—based on the sessions, they started noticing that there was a need for certain forms for us to have. For example, they said you know what? A lot of them don't really—don't know, like, any—like for example, their A1C, or they don't know the last time they had a physical. So, we—they said why don't we create some medical folders? So, with all these forms that we create, we put ‘em in folders. And a lot of ‘em actually take them to their doctor's visit. So, then the doctor can help them fill them out so they have those things for records. And there's even a page in the back that says things to remember to mention to my doctor. So, they can write things there. And they don't forget when they go see their doctor. So, it was a lot of things based on them seeing there was a need for, we started creating to help, you know, facilitate things”* (IW_2019_A).

#### 3.1.3. Training and evaluation

The community-based organization and research center team made several modifications to support CHWs/Ps delivering the program and their older participants, both of whom often have less formal education. During initial coaching calls to support PEARLS delivery after the team was trained in the model, the team walked through sample problem-solving treatment worksheets to emphasize having the participant drive each step—e.g., selecting possible solutions and weighing the pros and cons rather than CHWs/Ps skipping to offering and choosing solutions. The implementation team created a visual Stay Well Plan (a relapse prevention plan) to provide written reminders of skills learned, depression management strategies, and key social and health contacts in one place. In traditional PEARLS, this final session is done verbally without a form to leave behind with the participant. The visual Stay Well Plan provided specific activity prompts (physical, social, solo, spiritual, relaxation, other), space for medications and allergies, and names/contacts for family and friends and any support groups in addition to providers. The CHWs/Ps and their program manager also developed a structure for the follow-up phone calls after the in-person session ends; in the PEARLS manual, this is left unstructured which creates challenges for care managers without clinical training. The new structure provided focus on reinforcing depression monitoring, regular meaningful activity planning, and applying problem solving skills to overwhelming issues.

PEARLS training was not adapted since CHWs/Ps already were trained in other competencies that facilitate PEARLS delivery, such as community outreach and advocacy, service coordination, culturally competent communication, and health coaching and education (72). CHW/P already having these competences was recognized as facilitating PEARLS success “*since so much PEARLS is who is doing it*” [COACH_2019-08-23]. Providers recommended that future CHW/P PEARLS models integrate CHW/P and PEARLS training given the complementary skills needed for each. PEARLS technical assistance was adapted in several ways. Typically, PEARLS implementation includes monthly technical assistance coaching calls with providers across the country to support ongoing skill-building and collaborative problem-solving of issues as well as share successes and nurture a community of practice. For this project, coaching was provided more regularly by the UW focused on the implementation team (CHWs/Ps, program manager, LCSW clinical supervisor, and initially CBO leadership), moving from weekly to biweekly to monthly over the course of the 3-year grant. As described above, these initial weekly coaching calls provided an opportunity to strengthen skill-building in using the PHQ-9, and to discuss doing person-centered, structured PST and BA to support clients where they are. As the CBO built capacity for and confidence in delivering depression care, they presented at collaborative care model networking webinars and in-person meetings to share their expertise and wisdom with other clinical—community partnerships.

#### 3.1.4. Implementation strategies

Implementation strategies are approaches for facilitating CCM adoption and delivery, such as through capacity building and integrating the model into workflows. For PEARLS, usual implementation strategies include provider skills training, monthly practice coaching, regular clinical supervision, data registry for monitoring clients and treating-to-target (adjusting care as needed for persons who are not improving after several sessions), and participant engagement. These strategies were adapted in several ways by the community-based organization and research center team. While training was conducted as usual, coaching was modified to include more regular external facilitation that met weekly, then biweekly, finally moving to monthly coaching calls with the external practice coaches. Clinical supervision was adapted to include both (1) weekly internal facilitation where the CHW/P program manager and external practice coaches reviewed all cases, and (2) weekly clinical supervision with the psychiatric consultant and therapist that focused on a few complex cases rather than all new cases.

This adapted clinical supervision model provided in-depth consultation and support to address both participants' depression and other health and social needs, as well as support for the CHWs/Ps who were now being exposed to challenging circumstances by providing depression care in people's homes. In some cases, a participant would be flagged for discussion if their PHQ-9 scores were fluctuating. In other cases, discussion would be triggered by concerns about safety, elder abuse, medical misconduct, or other issues. For example, one support call discussed one participant's challenges with a caregiver who “*just drops off the food and leaves*” and a second person who had a broken glucometer and who was also experiencing mistreatment by staff at a medical clinic (CLIN_2019-10-16). Another call reviewed a case of a participant who was being exploited by a family member jeopardizing her housing placement but refusing to leave her home as it was a free place to live (CLIN_2018_05_02). As such, clinical supervision functioned to task-shift clinical work to CHWs/Ps who built their capacity for administering the PHQ-9 and delivering brief behavioral interventions (PST and BA), while bringing issues around medication and suicidality to the clinical supervision team. Furthermore, the team used a data registry which is used in collaborative care to systematically monitor and track all participants and follow-up with additional support to those who were not improving. The team used the registry's PHQ-9 graph of scores over time to illustrate participant's progress or fluctuations in symptoms based on external stressors like changes in health, housing, or care.

Engagement in depression care is essential for reaching older adults of color who have historically had poor access to care and cultural and social stigma about depression and depression care. Initially in 2015, the community-based organization planned to partner with a clinical setting to engage older adults in collaborative care, embedding their CHWs/Ps in the clinic site. However, as new partners they faced challenges getting buy-in from the clinic and receiving referrals for care. After trying unsuccessfully to partner with two different clinics, in 2017, they pivoted to the PEARLS model to provide more accessible care. As one research center team member described, “*the clinic has only 4 walls, community is more expansive*” [COACH_2017-07-14]. They then planned to work on clinical partnerships over the course of the PEARLS implementation. There is no one model for engaging PEARLS participants in care—the model is implemented by community-based organizations to reach older adults who are underserved, and they use whatever engagement strategies are appropriate for reaching these communities. In this case study, outreach was done in waves, which helped manage time and efforts at a resource-constrained organization like El Sol where outreach must be integrated into care delivery. For example, El Sol increased the frequency of their community presentations when they had a large number of clients graduating the program and thus had availability for new clients to be enrolled (PLAN_2018-06-07). Engagement occurred through healthy aging presentations at community centers and low-income senior housing sites in which fresh fruit and vegetable baskets were provided as incentives. Depression and emotional health were introduced as part of the presentation in a non-stigmatizing way, and PHQ-9 screening was done privately. The value of doing outreach this way to engage older adults who are underserved is shared by one member of the PEARLS implementation team.

“*[We] maybe do a presentation at a senior center. We make contact with those that are interested in the program. Then we ask if we can visit them in their apartment or the house. We develop a connection by just talking about different things. You know, the weather. Sometimes we do bring vegetables. We have connections with a local food distribution center that sometimes we're able to get vegetables, fruits. So, we give those to our potential clients. We tell ‘em what the program is about, how it's gonna benefit them. It's at no cost. And then we – of course, we listen to their stories. And once the clients feel – the patients feel that they can trust us, they're not difficult to work with. It's really about establishing that trust, making sure that they know we're there to help ‘em, not to take advantage of them like many people have done perhaps in their life. So, developing that trust in the beginning is critical. We've been able to work with them and them opening a door to us.… I think being available, being useful to them, really helps them feel they can trust us. And we don't do it just once. We do it, you know, several times so they can see that we're for real. We're there to help ‘em and support them as we work with the PEARLS Program. So, in general, those are the different strategies that we use”* (IW_2019_D).

#### 3.1.5. COVID-19 pandemic adaptations

With the onset of the COVID-19 pandemic, PEARLS delivery was moved primarily to phone or in some cases to videoconferencing when this did not present a barrier to access. The PEARLS team provided up-to-date COVID-19 information to assuage increased anxiety, and engaged family members to support older adults in addressing social and health needs (e.g., driving to get medications and food) as the CHWs/Ps were unable to play that role due to safety concerns. In some cases, the social and medical needs were exacerbated during the pandemic and the CHWs/Ps had to play a more active role to address acute needs rather than taking the time to empower older participants to address them: “*We have been changing in times [of] COVID…now we have to do certain things for them. Like for example, we call people, we call food centers to send food to these families. We tried to help. So it has been changing in terms of delivery, it's not just doing the service, but now we are in a pandemic that we have to do something else in order to help the community to survive, especially the seniors*.” While phone delivery worked well for the already engaged PEARLS participants, there was some concern with using the phone to engage new participants given many older adults are concerned about elder abuse and fraud and may not trust someone offering services by phone.

### 3.2. Determinants

Contextual determinants include both facilitators and barriers to PEARLS delivery, organized in this study by CFIR's six areas: intervention characteristics, inner setting, outer setting, implementation process, and system. CCM delivery was facilitated by its relative advantage, adaptability, and trialability; and the PEARLS team's collective efficacy, alignment with organization mission, and ongoing reflection and evaluation during implementation. Barriers to CCM delivery were challenges partnering with clinical partners for engagement, reimbursement and sustainment post-grant funding.

#### 3.2.1. Intervention characteristics

Providers, the organization and external stakeholders saw PEARLS as having a relative advantage over clinic-based depression care programs; PEARLS also provided a way to engage older adults specifically (much of El Sol's work focuses on children and families). PEARLS delivery demonstrated the value of engaging older adults in a community center that focused on reaching Latino children and families. Furthermore, the PEARLS model had perceived sustainability *via* reimbursement. PEARLS uses the PHQ-9 to identify and track participant outcomes which is used by health payors and clinical settings and aligns with recommended care for quality assurance and accountable care. The community-based organization's strong depression outcomes, CHW/P workforce, and reach of traditionally underserved older adults living with complex health and social needs demonstrated their value as care providers.

Initially, PEARLS had complexity that made it “*very time consuming*” to deliver. Some of this was serving older adults that need considerably more support than others to improve their depression, and some of this was the time needed due to the CHW/P team needing to adapt PEARLS for engaging older Latino immigrants with complex health and social needs. The ability for CHWs/Ps and the implementation team to adapt both PEARLS intervention and implementation through regularly trying out different tools and approaches helped them to counter its complexity with modifications that made the program work better for their organization, the providers, and the participants. Furthermore, CHWs/Ps also adapted their usual case management to address social needs using PEARLS Problem-Solving Treatment where the participant are empowered (e.g., contact health plan during the PEARLS session to change their primary care provider, get access to a freezer for home delivered meals). In this way, PEARLS adaptability and trialability helped to minimize complexity and facilitate delivery.

#### 3.2.2. Inner setting

The inner setting determinants were the most common type of determinants that emerged from data analysis. The culture (the norms, values, and mission) of both CHWs/Ps and community-based organizations set a strong foundation for PEARLS as a model for improving access to late-life depression care for older Latino immigrants. Both CHWs/Ps and community-based organizations bring experience, commitment, trust and relationships with the community—as one PEARLS team member shared, “*this is what we do, we are the sun for our community*” [the organization's name is the Spanish word for sun]. The collective efficacy of the PEARLS team was seen as instrumental for successful program delivery and ultimately participant health outcomes. All the team members—the CHWs/Ps, program manager, administrators, clinical supervisors—came together to adapt and deliver PEARLS well, achieving shared implementation and effectiveness goals. Team characteristics included strong buy-in to the model, experience with the community, willingness to go above and beyond and do whatever it takes, and clarity in their roles and responsibilities, which all helped nurture a learning climate to share power and ideas for ongoing quality improvement throughout PEARLS delivery. Lastly, the organization and providers had readiness for implementation including both available resources from grant support for on-going operations (training, staffing, time) and leadership engagement including commitment and involvement from both administrators and the program manager pre-implementation and during implementation.

The main inner setting barrier to PEARLS delivery was the ongoing challenges engaging with clinic partners. The original intent of the practice coaching support from UW in 2015 was to strengthen clinical-community partnerships to improve access to quality late-life depression care. Initially, the community-based organization aimed to partner with one clinic at a time to embed CHW/P-delivered collaborative care in their setting. When these clinic partnerships did not result in any referrals or engagement, the community-based organization adopted PEARLS to serve older Latino adults directly in their homes, hoping to continue partnering with clinics to receive referrals for the PEARLS program and refer older adults with poor access to primary care to these clinic(s). Time, trust, and turf issues hindered these linkages: the clinical and community settings each have different cultures and as such, the CBO indicated that two clinics they tried to partner with preferred to build their own program rather than refer out to CBOs. This means that even with strong depression outcomes, clinical-community partnerships may not transpire. Furthermore, resource-constrained CBOs like El Sol do not have available resources such as infrastructure to do the billing required to contract with clinics and receive a proportion of payment for services. As one PEARLS team member shared, *I'm not asking for the whole $75 that the insurance paid for; give us $50 and take the $25. And let us do the work, because the patient needs it....the clinics, they don't see us as a resource, sharing resources. Okay, if I'm getting $75, and somebody is doing a good job then I give them $50 to do it. I'd rather do it on my own, and that's what they are trying to do*. While the grant funding provided resources to build capacity to deliver integrated care, it did not provide the funding to address time, turf and trust issues or build needed infrastructure for sustaining program delivery.

#### 3.2.3. Implementation process

The process for PEARLS delivery included regular reflection and evaluation among team members, and engagement and shared decision-making both in planning for and throughout implementation. Although these activities were part of the CBO culture, they were also built into the PEARLS model of care in the form of regular clinical supervision to support front-line providers with limited formal education to deliver depression care. Regular coaching calls provided opportunities to share successes, problem-solve issues, and nurture a community of practice. Additionally, group supervision and coaching calls were valued for bringing together people with similar work and passions for helping older communities and getting ideas to try out with clients. These implementation processes were seen as instrumental for both implementation and effectiveness, with team members coming together to support both older adults and each other and plant seeds for future health and wellbeing.

“*Having a team to be able to discuss cases on a weekly basis has truly contributed not only to the program's success, but to the patients getting better as well. It truly takes a village to help patients overcome their depression, especially when this includes navigating through the health care system. When everyone's background and expertise are put together, El Sol has been able to accomplish so many wonderful things. Yes, the PHQ-9s do drop significantly and there are a lot of patients that have completed PEARLS but hearing and seeing how happy patients are, how thankful they are for having received PEARLS is truly priceless. Knowing that seniors are not only reducing their depression but are educating themselves to be able to be independent once again is extremely valuable”* [PROG_Q4_2018].

The CHW/P PEARLS team used weekly clinical supervision sessions with their manager and external clinical supervisors, and regular technical assistance calls with external practice coaches to support both the participants and the CHWs/Ps. A team member described how external supervision was helpful to connect with subject matter experts who had practical experience working with the community: *It's been wonderful…just very, very helpful. They have so much knowledge of the program that during our phone calls we would talk about issues we're having with clients and they would always give us great advice. I feel like they had a good understanding of the type of population that we're working with, with Latinos, and understood the struggles that they go through, the challenges that they face. So I think they were always able to give us great guidance* [IW_2020_B]. The team recognized that this support of CHWs/Ps and their efforts was essential to be able to provide PEARLS in marginalized communities given the “*horror stories that have passed in their life*” (IW_2020_C).

#### 3.2.4. Outer setting

External incentives from the grant supporting the PEARLS implementation was key for the community-based organization to adopt and deliver PEARLS. External policy levers such as the U.S. Department of Labor including CHWs/Ps in their Occupational Outlook Handbook and the Affordable Care Act also facilitated PEARLS adoption and delivery as they offered opportunities post-grant support to resource-constrained organizations who recognize the need to plan for sustainability before they adopt a program [PROG_2017_Feb]. However, despite efforts to sustain the effective model post-grant funding, these external drivers were inadequate to sustain the program after the grant ended. Community characteristics both facilitated and challenged PEARLS delivery in the older Latino immigrant community. The lack of culturally and linguistically appropriate mental health services created a demand for PEARLS, while stigma about depression and depression care and scams geared toward older adults meant at times it took longer to engage participants. Widespread poverty, elder abuse and discrimination, poor quality health care, and lack of transportation, insurance, and resources challenged program delivery and engagement even though participants' PHQ-9 depression improved over the course of PEARLS. At the end of the day, multi-level interventions that tackle structural inequities are needed to support both implementation and effectiveness of a largely individual-focused model like PEARLS.

#### 3.2.5. System

There was strategic policy alignment with the PEARLS model and current recommendations for improving access to quality mental health care. Specifically, this alignment is to build capacity among front-line providers to task shift and task share care in accessible and appropriate community settings that reach older adults who have been underserved. However, the current systems architecture that still prioritizes clinic-based care (even by clinic CHWs/Ps) and a lack of resource continuity after grant funding means that the CHW/P PEARLS model based at a community-based organization is not currently adequately or sustainably covered. Furthermore, external funding agent priorities both helped and hindered PEARLS delivery. On the one hand, it provided essential resources for PEARLS to be done by community-based CHWs/Ps to engage older Latino immigrants in care through a model that integrated with their CHW/P role and responsibilities and provided additional behavioral health training and clinical support. However, since the community-clinical partnerships externally created were prompted by the grant rather than organic, funding an integrated care model did not result in true (or fully integrated) partnership. One of the PEARLS implementation team members captured this tension in an interview.

“*I love the model of integrating because it's powerful. I think we need to work together. But forcing us to kind of, this structure, put us in a hard situation because maybe they are not ready or maybe the city is not ready, or maybe they don't understand the why behind the structure or the form. In my opinion, I would do it differently so that we come up to our own best way to do partnerships. And you guys oversee the process, I mean whoever is going to be commanding the project or evaluating the project. And at the end, it's not about if I learn to work with another or with the clinic, it's at the end, are we still working together after the grant leaves....they are released, now we finish, you guys do your thing, I do my thing, or did we really integrate systems?”* [IW_2019_C].

### 3.3. Implementation outcomes

Participation data (quantitative) suggests PEARLS was acceptable, feasible and done with fidelity (Table 5). Participants had mean (SD) 6.6 (2.1) home visits and 2.9 (0.9) phone visits, aligning with the PEARLS model of six to eight home visits and three follow-up calls. The PHQ-9 was used both for identifying persons living with clinically significant depression, as well as monitoring treatment over time. Each participant had on average 9.8 (2) contacts. While not a requirement of the PEARLS model, one-third of participants (35.6%) completed at least one session with a family member, friend, or home health aide, and 13.4% completed at least 3 sessions with this support. Seventy-three percentage of participants were reviewed at least once during clinical supervision meetings.

Analysis of qualitative data provides additional evidence illustrating PEARLS strong early-stage implementation outcomes. Looking at acceptability, participants, providers, and the organization highlighted the strong trust and rapport that CHWs/Ps have with older Latino adults who have been underserved by other care systems. Offering services in Spanish and understanding the cultural context of being Latino (e.g., being Mexican-American, living as an immigrant in the U.S.) influenced engagement and impact. When older adults are first enrolled in PEARLS, the CHWs/Ps ask the participants where they were originally from and how long they had been in this country to help the PEARLS team understand how they could best support them. For example, someone who has been in the U.S. for thirty or more years may consider the US their home, whereas someone who arrived to the U.S. a year ago may be feeling homesick and may not have a strong support system yet. For older Latina women, PEARLS helped support self-care, for example by providing encouragement to put their needs before adult children who were sometimes taking advantage of them, while working within cultural values of *familismo*—commitment and loyalty to family. For older Latino men, PEARLS offered a place where they could tackle problems and gain comfort talking about their feelings. Providers felt that the program improved their skills for supporting older adults so that they could take better care of their depression and their lives. Though most participants were very satisfied with PEARLS, acceptability was lower for older adults with lower motivation or who were not ready to change (as suggested by qualitative participant and provider data; motivation or readiness was not measured quantitatively). Furthermore, high acceptability did not always translate into effectiveness as is theorized in the Implementation Outcomes Framework (67); while depression improved across participants, significant stressors remained that PEARLS is not sufficient to address (e.g., poor quality health care and caregiving that were out of participant's or provider's control): “*some problems we can solve, others we cannot*” [IW_2019_E].

The CHW/P PEARLS model was highly feasible. Going into the home was key for providing depression care given stigma about asking for help and receiving care, and challenges accessing clinical care such as lack of insurance and transportation. The community-based organization was already providing services out in the community so going into the homes was natural for CHWs/Ps, though in some cases they had to make sure the neighborhood or home environment was safe for both them and the program participant. In addition, PEARLS fit well with the CHW/P model of care as it built capacity among community members to provide culturally and linguistically appropriate depression care to older Latino immigrants, taught them PST and BA tools to increase older adults' health knowledge and self-sufficiency, and facilitated access to other services to address chronic health conditions and acute social needs. Lastly, feasibility was also enhanced by building self-care of CHWs/Ps into program management and clinical supervision, as described above in the implementation processes section. With CHWs/Ps, the community-based organization “*recruits hearts and trains brains*” [IW_2020_A]. This sentiment means that CHWs/Ps need support for self-care as they often extend themselves to support fellow community members (e.g., share their own food). That said, it is this heart that makes CHWs/Ps so impactful in their work as one cannot train hearts but can train brains to integrate PHQ-9s and PST and BA into their work.

Initial training plus ongoing external coaching facilitated the strong collaborative implementation team delivering PEARLS with fidelity. The coaching helped to hone CHWs/Ps skills doing routine PHQ-9 assessment as a way to provide psychoeducation about depression symptoms and adjust care if symptoms were not improving. It also helped integrate person-centered, structured behavioral interventions like PST, and BA into home and phone visits. While the CHW/P PEARLS model had key ingredients for sustainability (successful implementation *via* CHW/P workforce that engages older Latino adults who are underserved, strong depression outcomes and addressing social needs) it was not sustained due to challenges with referrals and reimbursement *via* clinical partnerships (e.g., resources required for community-based organizations to meet health payors requirements for billing). For the CHW/P PEARLS model to be sustained, there also needs to be consideration for the collective efficacy of a trained team working together for 3 years which could not be immediately replicated with new staff.

### 3.4. Effectiveness

Older Latino adults began PEARLS with a PHQ-9 of 16.6 (3.6) indicative of moderate depression severity (Table 6). Older Latino adults significantly improved their depression by an average of 13.7 (4.4) from PEARLS enrollment to post-intervention period (typically 5 months following enrollment), with the paired t-test of pre-post PHQ-9 scores, *t* = 37.9, *p* < 0.001). Over the course of the 6-month intervention period, the majority of PEARLS participants significantly improved their depression: 98.0% improved their PHQ-9 score by 50% or more (clinical response), 85.9% had a PHQ-9 <5 (suggesting remission from clinically significant depressive symptoms), and 98.7% had a 5-point or greater improvement from baseline PHQ-9 scores.

**Table 6:** PEARLS depression outcomes (*N* = 149)^a^.

	***n* (%)**
Baseline PHQ-9, *mean (sd)*	*16.6 (3.6)*
PHQ-9 ≥ 50% improvement (response)	146 (98.0)
PHQ-9 last score <10	145 (97.3)
PHQ-9 last score <5 (remission)	128 (85.9)
PHQ-9 ≥ 5+ point improvement	147 (98.7)
PHQ-9 change, baseline to last score, *mean (sd)*	*13.7 (3.9)*
PHQ-9 % change from baseline to last score, *mean (sd)*	*82.2 (15.9)*

a PHQ-9 data is available for people with at least one session following enrollment (149/152, 98%).

All data are presented as *n* (%) except when displayed in italics which denotes data is presented as mean (sd).

In addition to significant improvements in depression, 246 health education materials were shared to support chronic disease management and 393 referrals to address social needs were made over the course of PEARLS (on average 2.6 social needs per participant) (Table 5). Eighty percent of referrals resulted in connections to services, and 10% of referrals were for services the participants were already connected to. The most common referrals were for resources for better quality health care, family and social support, housing, legal aid, immigration and citizenship supports, in-home support services and caregiver, transportation, and spirituality. These social needs referrals were seen as a participant outcome, as well as a driver for improving depression and a way for participants to feel heard and seen, and thus, stay engaged with PEARLS.

Qualitative data analysis suggested several possible mechanisms by which PEARLS improved depression as well as other health and social impacts. One participant during an interview (IW_E) described what she referred to as her “Cinderella” story. The participant was an older, 75-year-old, Latina who was living with disabilities after recently surviving a stroke. She was experiencing a difficult living situation with her daughter who treated her like a maid, making her cook and clean for room and board. Over the course of PEARLS, she and her CHW/P coach used problem solving and behavioral activation to get on social security benefits and move into a senior apartment. These actions helped her to improve her quality of life, feel more independent, and get engaged in pleasurable activities. In addition to changing their behaviors and context, PEARLS participants also changed their attitudes and beliefs that had contributed to their depression—one PEARLS team member [IW_2020_C] indicated that participants often shared “*Oh, I feel better because I don't worry about all the things that bothered me*” which helped their PHQ-9 do down.

## 4. Discussion

This case study describes adapting PEARLS, a home-based collaborative care model, to improve access to quality depression care for older Latino immigrants who have been traditionally underserved by health care. As summarized in Figure 2, PEARLS was adapted for impact by building capacity among the existing workforce to expand access to late-life depression care: CHWs/Ps were trained as CCM care managers, and a psychiatric consultant and licensed clinical social worker provided clinical supervision for older adults with complex health and social needs. CHWs/Ps integrated chronic disease management and stress management tools into existing PEARLS content (Problem-Solving Treatment and Behavioral Activation) to address participants' poor access to health care and to help manage multiple life stressors. Embedding depression screening into low-income housing sites and providing care in the home and by phone further improved engagement and access to care. Regular training, coaching, clinical supervision, and data monitoring supported PEARLS delivery in this new context.

Before and during PEARLS implementation, the internal implementation team at the community-based organization and external coaching team at the applied research center partnered to make fidelity-consistent adaptations that aligned with core functions (73) of the PEARLS model. Though largely unplanned and reactive, these adaptations functioned as an implementation strategy for health equity (45) to improve program fit for participants, providers, organization, and context. This case study suggests that adapting PEARLS for delivery *via* CHWs/Ps was effective for addressing depression among older Spanish-speaking Latino immigrants with complex health and social needs. A 98% depression response rate is almost double what is seen in the 54% benchmark for collaborative care in a clinic setting (74), and what was found in the original PEARLS effectiveness trial (43% response rate) (25).

Some of this significant improvement may stem from PEARLS participants having on average 2.6 referrals for social needs which drive both access and outcomes. While PEARLS is not a social needs intervention *per se* (75), both the chronic care model and collaborative care model call for linking participants to additional services and supports to improve their depression outcomes. Our findings about the importance of addressing unmet health and social needs (e.g., caregiving to support activities of daily living, and linking to better quality primary care or augmenting current primary care through chronic disease management) to enhance depression outcomes aligns with findings from a recent related study that suggested the collaborative care model integrated with clinical and community care can strengthen depression care for older adults (76). To address health inequities among older adults who have been underserved, social needs may need to be more explicitly screened for, intervened upon, and monitored as part of depression care interventions both as a mechanism for improving depression outcomes and as a way to keep participants engaged.

This adapted PEARLS model had high acceptability, feasibility and fidelity with a population for whom depression and depression stigma has been well-established (77). PEARLS patient-centered model may have also played a role by addressing things that matter to older adults rather than being interventionist directed. In other words, the program could be socially valid and culturally appropriate (64) while addressing fundamental drivers of health disparities like lack of access to resources and power as well (78). While clinical effectiveness may still be the metric on which both health and SDOH intervention studies are measured (given the centrality of health) (79), addressing outcomes that matter to older adults fits with the Patient Centered Outcomes Research Institute (PCORI) and other efforts to center outcomes that matter to populations who have been underserved (80). These factors also appeared to improve PEARLS engagement and fit to a resource-constrained context for better equity in access and outcomes (81).

Underlying adaptations, implementation and outcomes were multi-level contextual determinants. Inner setting determinants such as collective efficacy (team buy-in, clarity in roles, share power, and ideas for quality improvement), an implementation team with long-term, ingrained partnerships with the community, and leadership buy-in and commitment, alongside implementation process determinants (regular reflection and evaluation, engagement and shared decision-making, *via* regular coaching calls with external facilitators and internal team meetings with CHWs/Ps and with clinical supervisors) appeared to drive program delivery and impact. Indeed, prior to adopting a home-based CCM model (PEARLS), the community-based organization and external coaches had unsuccessfully tried to engage with clinical partners for clinic-based CCM and ran into issues with time, turf, and trust (82).

System of care challenges remain for resource-constrained settings to sustainably deliver PEARLS. For instance, while PEARLS participants addressed social and health needs through behavioral interventions and linkages to care, access to quality health and social care remains lacking for some older Latino immigrant communities. Likewise, while new funding and delivery mechanisms such as Accountable Communities of Health emphasize value over volume of care and prioritizing populations underserved rather than one-size-fits-all, these models have not translated into sustainable funding for CHWs/Ps and community-based organizations. As such, one-on-one community-based interventions like PEARLS will have limited impact and reach unless policy, environmental, and systems interventions address these structural drivers of health inequities.

While we chose a case study for its pragmatic design and community-engagement, this design did not include a randomized control group. Thus, it has several limitations. First, as single-group design, we are limited by threats to internal validity (such as selection bias) and we cannot make causal inferences about PEARLS effectiveness or implementation; other unmeasured confounding factors may have influenced program impact or delivery. This case study found very high depression response rates which are important to try and replicate in a different CHW/P setting serving older Latinos. Future hybrid studies (83) are needed that include context as an independent variable in addition to intervention and implementation strategies. Specifically, future hybrid studies should measure and evaluate the implementation-related effects of variations in the contextual determinants identified by this case study, to both further health equity research and implementation science and to optimize PEARLS delivery in real-world contexts for older Latino immigrants and organizations that engage them (84).

Prospective studies would also need to address the limitation that as a secondary data analysis, our data sources were not collected for research but rather as part of routine program delivery, monitoring, and evaluation. This approach made the data collection more pragmatic and allowed us to incorporate multiple data sources and diverse perspectives, but future research would allow specific measurement of possible unmeasured confounders identified in this case study. Future prospective, longitudinal data collection would also allow us to tease out the contribution of social needs referrals to depression outcomes, such as whether these referrals led to lower unmet social needs which in turn reduce depressive symptoms directly or indirectly *via* better access to health care. Lastly, we recognize the limitations in choosing to adapt an existing EBP (PEARLS) rather than develop and test a new EBP in partnership with the community-based organization and older adults. Recommendations for more equitable implementation specifically call out the need for more grassroots approaches that “examine community realities from the outset, along with root causes of the needs and barriers an intervention seeks to address, including historical and structural racism [and] involve the people most at stake in the program in selecting programs, policies, and approaches that will be relevant to their communities” [(85), p. 4].

In conclusion, this case study demonstrates the value of engaging CHWs/Ps at community-based social service organizations to improve access to quality depression care for older Latino adults. Specifically, training valued community members to provide care, providing ongoing external and internal coaching and consultation to support both CHWs/Ps and care delivery, and addressing health social needs as part of depression care strengthened the equitable implementation of PEARLS for older Latinos and reduced disparities in both access to care and depression outcomes. Providing culturally and linguistically appropriate care through trusted providers in accessible settings was essential for improving program fit and impact on depression and upstream social outcomes.

## Data availability statement

The data analyzed in this study is subject to the following licenses/restrictions: raw data will be made available on reasonable request with any data that may risk loss of confidentiality redacted. Requests to access these datasets should be directed to LS, lesles@uw.edu.

## Ethics statement

Ethical review and approval was not required for the study on human participants in accordance with the local legislation and institutional requirements. Written informed consent for participation was not required for this study in accordance with the national legislation and the institutional requirements.

## Author contributions

LS conceived of the study, served as a practice coach, led the analysis, and drafted the manuscript. AG, RP, AH, and AF adapted and delivered the intervention and contributed to manuscript review and editing. TH, MV, and JU made intellectual contributions to study development, advised on intervention adaptations, implementation and evaluation, and contributed to manuscript review and editing. PR made intellectual contributions to study development, served as a practice coach, advised on intervention adaptations, implementation and evaluation, and contributed to manuscript review and editing. SH and LH made intellectual contributions to study development, led the parent project qualitative evaluation, and contributed to manuscript review and editing. LR advised on project implementation and evaluation and contributed to manuscript review and editing. All authors contributed to the article and approved the submitted version.
